# Antimicrobial resistance patterns of bacteria isolated from dogs with otitis

**DOI:** 10.1017/S0950268818003278

**Published:** 2019-03-08

**Authors:** C. Bourély, G. Cazeau, N. Jarrige, A. Leblond, J.Y. Madec, M. Haenni, E. Gay

**Affiliations:** 1 École Nationale des Services Vétérinaires, ENSV, VetagroSup, Marcy l’Étoile, France; 2 Université de Lyon, ANSES, Laboratoire de Lyon, Unité Epidémiologie et Appui à la surveillance, 31 avenue Tony Garnier, 69007 Lyon, France; 3 EPIA, UMR 0346, Epidémiologie des maladies Animales et zoonotiques, INRA, VetAgro Sup, University of Lyon, F-69280, Marcy L'Etoile, France; 4 Université de Lyon, ANSES, Laboratoire de Lyon, Unité Antibiorésistance et Virulence Bactériennes, 31 avenue Tony Garnier, Lyon 69007, France

**Keywords:** Antimicrobial resistance, canine otitis, multidrug resistance, RESAPATH, time series

## Abstract

Antimicrobial-resistant bacteria in dogs can be transmitted to humans and close contact between dogs and people might foster dissemination of resistance determinants. The aim of our study was to describe the antimicrobial resistance (AMR) pattern of the major causative agents of canine otitis – one of the most common diseases in dogs – isolated in France. Data collected between 2012 and 2016 by the French national surveillance network for AMR, referred to as RESAPATH, were analysed. Resistance trends were investigated using non-linear analysis (generalised additive models). A total of 7021 antibiograms were analysed. The four major causative agents of canine otitis in France were coagulase-positive staphylococci, *Pseudomonas aeruginosa*, *Proteus mirabilis* and streptococci. Since 2013, resistance to fluoroquinolones has been on the decrease in both *P. aeruginosa* and *Staphylococcus pseudintermedius* isolates. For *P. aeruginosa*, 19.4% of isolates were resistant to both enrofloxacin and gentamicin. The levels of multidrug resistance (acquired resistance to at least one antibiotic in three or more antibiotic classes) ranged between 11.9% for *P. mirabilis* and 16.0% for *S. pseudintermedius.* These results are essential to guide prudent use of antibiotics in veterinary medicine. They will also help in designing efficient control strategies and in measuring their effectiveness.

## Introduction

Dogs are considered to be one of the potential reservoirs of antimicrobial resistance (AMR) determinants that can be transmitted to humans through direct or indirect contact [1, 2]. The number of people living with pets, especially dogs, has been increasing worldwide over the last few decades [3, 4]. In France, the estimated dog population has been stable since 2012 at around 7.3 million, and about 20% of households in the country accommodate pets [4]. As the selection and spread of AMR has serious consequences for human beings, knowledge of the prevalence of resistance and temporal variations, with regular updates, in both humans and animals, is required to assess the potential threats to public health, to design efficient control strategies, and to measure their effectiveness.

Canine otitis is one of the most common diseases in dogs. Bacteria and yeast are considered to be both predisposing and perpetuating factors [5]. In France, otitis is usually treated empirically at point of diagnosis, with cleaning, administration of antibiotics and topical anti-inflammatory drugs. The choice of antibiotic is based on otoscopic examination of the ear canal, cytological results, the clinical experience of veterinarians and therapeutic guidelines. In particular, the therapeutic choices take into account the spectrum of activity of antibiotics, their administration route and their availability. In France, nine antibiotic classes are recommended and are available on the market for the treatment of canine otitis: penicillins, cephalosporins, aminoglycosides, folate pathway inhibitors, macrolides, phenicol, fusidanin and polymyxins are first-line treatment, whereas fluoroquinolones are recommended as second-line treatment [6]. Even if guidelines recommend prescribing these antibiotic classes, no distinction is made according to the pathogens involved. In this context, knowledge of the resistance to these antibiotic classes of the most common bacteria involved in otitis could greatly help in selecting the appropriate antibiotics and provide a scientific basis for treatment guidelines of dog otitis in France. Indeed, French guidelines point out the lack of French data available on the resistance of bacteria involved to better guide prudent initial prescription [6].

The most common ear pathogens isolated from dogs are coagulase-positive staphylococci (*Staphylococcus pseudintermedius*) and *Pseudomonas aeruginosa* [5, 7]. So far, only few studies conducted in other countries have investigated the resistance of pathogenic strains isolated from canine otitis and they mainly concerned *S. pseudintermedius* [7–9]. The number of isolates was often limited [7–12], and studies mixed samples collected from the skin, ear or soft tissues [13–16], or gathered samples from sick and healthy dogs [17] to increase the sample size. In addition, these investigations were mainly local (collecting data from one or few practices or data from particular administrative region, e.g.). Therefore, the scope of the results obtained is limited and generalisation to other countries is not possible. In addition, it would be necessary to investigate resistance variations over time to detect potential emergences and assess the efficacy of control strategy. Only one study has explored AMR trends in dog otitis so far, but this work mixed samples from dogs and cats throughout Europe and only linear regression was used to analyse results from 2002 and 2009 [18].

The aim of our study was to characterise the AMR patterns of the most frequent bacterial causative agents of canine otitis isolated in France, and to describe their temporal variations from 2012 to 2016. This study was performed using the dataset from the well-established French national surveillance network for AMR in pathogenic bacteria from animals, referred to as RESAPATH [19], which collects antibiograms, thanks to its laboratory members which are implanted in all administrative regions in France.

## Materials and methods

### Source of data

This retrospective study was performed using data from the RESAPATH laboratories. Created in 1982, RESAPATH has expanded to include all animal species, including dogs since 2007. It collects results from antibiograms performed by French veterinary laboratories participating in the AMR surveillance network. These antibiograms are initially requested by veterinarians in a context of disease for diagnostic purposes, such as canine otitis. All laboratories perform antibiograms by the disk diffusion method, according to the recommendations of the Antibiogram Committee of the French Society of Microbiology (CA-SFM), and inhibition zone diameters are then compiled in the RESAPATH database. From this database, we extracted data concerning the most frequent bacterial genera isolated from dogs with clinical otitis from 2012 to 2016. Variables extracted included bacterial species, occurrence of clinical otitis, sampling date, administrative region of the sampling and inhibition zone diameters to each tested antimicrobial agent. For the bacterial genera considered, we selected appropriate antibiotics of relevance in veterinary and human medicine; antibiotic were selected according to their spectrum of activity against the pathogen considered, their use to treat canine otitis (topical and systemic antibiotics), their public health interest including critically important antibiotics (fluoroquinolones and third-generation cephalosporins) and their use as specific indicators of resistance (e.g. cefoxitin and cefovecin for the detection of phenotypically methicillin-resistant staphylococci).

### Data analysis

Among each bacterial genus, we performed an analysis at the scale of the bacterial species if the number of isolates collected from this bacterial species was above 1000, and otherwise at the genus scale. The first step in the analysis was to categorise isolates as susceptible (S), intermediate (I) or resistant (R), using their inhibition zone diameters compared with the breakpoints recommended by the veterinary section of the CA-SFM. From an epidemiological point of view, the event of interest is the non-susceptibility to a particular antibiotic, indicating that the isolate is no longer a wild-type strain. For this reason, intermediate isolates were grouped together with resistant isolates in the non-susceptible population, referred to as resistant in this paper.

For each bacterial species (or genus)/antibiotic combination, the indicator of resistance was defined as the ratio between the number of resistant isolates and the total number of isolates tested at each time step. To model changes in AMR pattern, we used generalised additive models (GAMs) [20]. GAM extends traditional generalised linear models by replacing the linear predictor with unspecified non-parametric functions. GAM is a flexible and effective technique for conducting non-linear regression analysis in time-series studies. Here, GAM models were used to decompose raw signal (variation of resistance levels over time) and to capture trends and seasonality. Time-series analyses were performed for bacterial species (or genus)/antibiotic combination for which we had at least 25 antibiograms per time step [21]. Using count data, the bimonthly number of resistant isolates was modelled with a binomial negative regression with an offset equal to the log of the total number of isolates submitted bimonthly. The GAM models were structured and analysis performed as described by Boireau *et al*., using a separate and independent GAM for each bacterial species (or genus)/antibiotic combination [22]. The models presented in this paper included non-parametric smooth functions of calendar time designed to control for trend and seasonality. Smoothing parameters were estimated using cross-validation. We considered a *P*-value of ⩽0.05 as a statistically significant difference. If trend variations were not significant, the trend was stationary. R, version 3.4.3 was used for all statistical analyses (gamm4 package for GAM implementation).

We carried out multidrug resistance (MDR) analyses according to the conventional definition: isolates with acquired resistance to at least one antibiotic in three or more antibiotic classes [23]. We considered only the antibiotic classes most frequently tested by veterinary laboratories; the phenicol and fusidanin classes were not taken into account in the MDR analysis. We estimated the proportions of isolates that were pan-susceptible (susceptible to all classes of antibiotics tested), resistant to one or two antibiotics from different classes, MDR and resistant to all classes of antibiotics considered. Confidence intervals (CI) around the proportion estimates were calculated using the exact binomial method (*P*-values were two-sided).

## Results

### Bacteria isolated from dogs with otitis

The most frequent bacterial genera isolated from dogs with otitis from 2012 to 2016 in France were: coagulase-positive staphylococci, *Streptococcus* spp., *Pseudomonas* spp. and *Proteus* spp. The distribution among bacterial genera and species is presented in Table 1. Considering the number of isolates, we studied *S. pseudintermedius* (formerly *Staphylococcus intermedius)*, *P. aeruginosa* and *Proteus mirabilis* at the species level. Considering the zoonotic potential of *Staphylococcus aureus* [13–15], and the importance of this pathogen in nosocomial infections in both human and veterinary medicine [24, 25], we also performed analysis on this bacterial species, in spite of the limited number of isolates collected over the period. Streptococci were analysed at the bacterial genus level. On the basis of the extracted data (7623 isolates in total, Table 1), 33.0% of the antibiograms concerned *S. pseudintermedius*, 3.9% *S. aureus*, 27.5% *P. aeruginosa*, 13.6% *P. mirabilis* and 14.1% streptococci (Table 1). A total of 7021 isolates regarding *S. pseudintermedius*, *S. aureus*, *P. aeruginosa*, *P. mirabilis* and streptococci collected by RESAPATH between 1 January 2012 and 31 December 2016 were analysed in this study. Considering the bacterial genera identified, we selected the antibiotics presented in Table 2 for further analyses.

**Table 1.** Number of isolates collected from dogs with otitis between 2012 and 2016 by genus and bacterium identified

Genus	Bacterial species	Number of isolates	Proportion of isolates (%)	Total number of isolates per genus
Coagulase-positive staphylococci	*Staphylococcus pseudintermedius*	2513	33.0	3133
	*Staphylococcus aureus*	294	3.9	
	Other	326	4.3	
*Pseudomonas* spp.	*Pseudomonas aeruginosa*	2103	27.5	2315
	Other	212	2.8	
*Proteus* spp.	*Proteus mirabilis*	1039	13.6	1103
	Other	64	0.8	
*Streptococcus* spp.	*Streptococcus canis*	447	5.9	1072
	Other	625	8.2

**Table 2.** Antibiotics tested and the corresponding antibiotic classes for each bacterial genus

Bacterial genus	Antibiotic	Antibiotic class
Coagulase-positive staphylococci	Gentamicin	Aminoglycosides
	Cefoxitin or cefovecin	Cephalosporins
	Enrofloxacin	Fluoroquinolones
	Trimethoprim-sulfamethoxazole	Folate pathway inhibitors
	Fusidic acid	Fusidanin
	Erythromycin	Macrolides
	Penicillin G	Penicillins
	Chloramphenicol	Phenicol
*Pseudomonas* spp.	Gentamicin	Aminoglycosides
	Enrofloxacin	Fluoroquinolones
*Proteus* spp.	Gentamicin	Aminoglycosides
	Ceftiofur	Cephalosporins
	Enrofloxacin	Fluoroquinolones
	Trimethoprim-sulfamethoxazole	Folate pathway inhibitors
	Amoxicillin,	Penicillins
*Streptococcus* spp.	Gentamicin	Aminoglycosides
	Enrofloxacin	Fluoroquinolones
	Trimethoprim-sulfamethoxazole	Folate pathway inhibitors
	Erythromycin	Macrolides
	Chloramphenicol	Phenicol
	Oxacillin	Penicillins

### Levels of resistance over the entire period 2012–2016

Over the studied period, resistance to penicillin was high for staphylococci (68.5% (66.6–70.3) for *S. pseudintermedius*, 70.9% (65.1–76.3) for *S. aureus*), whereas it was lower for *P. mirabilis* (28.9% (26.1–31.9)) and streptococci (14.4% (12.0–17.1)) (Table 3). The level of resistance to ceftiofur was low for *P. mirabilis* (2.4% (1.6–3.6)). Concerning resistance to cephalosporins in Gram-positive isolates, 9.4% (7.8–11.2) of *S. pseudintermedius* were resistant to cefovecin (phenotypically methicillin-resistant *S. pseudintermedius –* MRSP) and 10.6% (7.0–15.1) of *S. aureus* were resistant to cefoxitin (phenotypically methicillin-resistant *S. aureus –* MRSA). The levels of resistance to erythromycin and chloramphenicol were between 25% and 40% and were similar for *S. pseudintermedius*, *S. aureus* and streptococci. The resistance to trimethoprim-sulfamethoxazole ranged from 10.2% to 22.9% for the studied bacteria. The level of resistance to gentamicin was low for *Streptococcus* spp. (3.3% (2.2–4.8)), higher for *P. mirabilis* (10.3% (8.5–12.3)), *S. aureus* (12.9% (9.2–17.5)) and *S. pseudintermedius* (13.5% (12.2–14.9)), and reached 17.9% (16.3–19.6) for *P. aeruginosa*. Finally, the level of resistance to fluoroquinolones was below 15% for *P. mirabilis* and staphylococci isolates, but higher for *Streptococcus* spp. (62.9% (59.8–65.9)) and *P. aeruginosa* (67.7% (65.6–69.8)).

**Table 3.** Resistance of isolates from dogs with otitis in France over the period 2012–2016

Bacterium	Antibiotic	Number of resistant isolates	Total number of isolates	Level of resistance (%) with 95% CI	Resistance trend from GAM model
*Staphylococcus pseudintermedius*	Penicillin G	1650	2409	68.5 (66.6–70.3)	Non-linear
	Cefovecin	112	1187	9.4 (7.8–11.2)	–
	Erythromycin	716	2401	29.8 (28.0–31.7)	Stationary
	Gentamicin	337	2491	13.5 (12.2–14.9)	Stationary
	Enrofloxacin	274	2115	13.0 (11.6–14.5)	Non-linear
	Fusidic acid	108	1757	6.1 (5.2–7.4)	–
	Chloramphenicol	196	700	38.9 (34.6–43.3)	–
	SXT^a^	297	2432	12.2 (10.9–13.6)	Stationary
*Staphylococcus aureus*	Penicillin G	190	268	70.9 (65.1–76.3)	–
	Cefoxitin	26	246	10.6 (7.0–15.1)	–
	Erythromycin	80	265	30.2 (24.7–36.1)	–
	Gentamicin	36	278	12.9 (9.2–17.5)	–
	Enrofloxacin	32	267	12.0 (8.3–16.5)	–
	Fusidic acid	17	148	11.5 (6.8–17.8)	–
	Chloramphenicol	37	119	31.1 (22.9–40.2)	–
	SXT^a^	28	274	10.2 (6.9–14.4)	–
*Streptococcus* spp.	Oxacillin	112	778	14.4 (12.0–17.1)	–
	Erythromycin	253	1021	24.8 (22.2–27.5)	–
	Gentamicin	26	790	3.3 (2.2–4.8)	–
	Enrofloxacin	627	997	62.9 (59.8–65.9)	–
	Chloramphenicol	89	252	35.3 (29.4–41.6)	–
	SXT^a^	210	1013	20.7 (18.3–23.4)	–
*Pseudomonas aeruginosa*	Gentamicin	375	2096	17.9 (16.3–19.6)	Stationary
	Enrofloxacin	1352	1996	67.7 (65.6–69.8)	Non-linear
*Proteus mirabilis*	Amoxicillin	284	982	28.9 (26.1–31.9)	–
	Ceftiofur	24	987	2.4 (1.6–3.6)	–
	Gentamicin	106	1034	10.3 (8.5–12.3)	–
	Enrofloxacin	134	1013	13.2 (11.2–15.5)	–
	SXT	232	1015	22.9 (20.3–25.6)	–

a Trimethoprim-sulfamethoxazole; ‘–’: no analysis performed (<25 isolates per time step).

### AMR trends

**Fig. 1.**
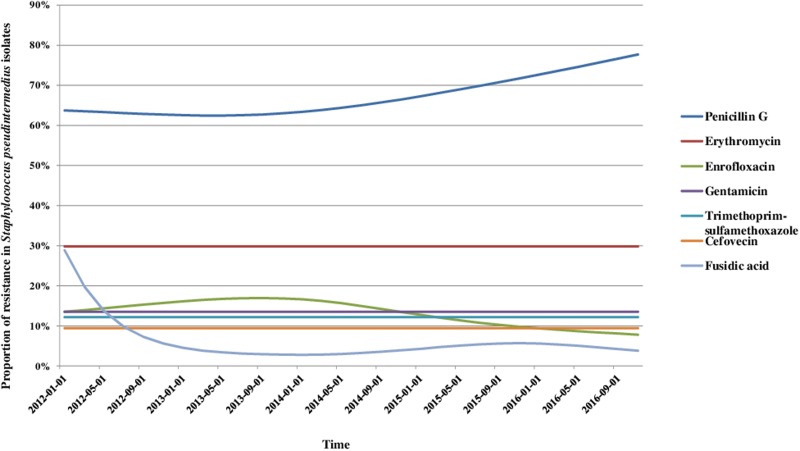
Trends for antimicrobial resistance in *Staphylococcus pseudintermedius* isolates from dogs with otitis during the period 2012–2016, on a bimonthly time step.

**Fig. 2.**
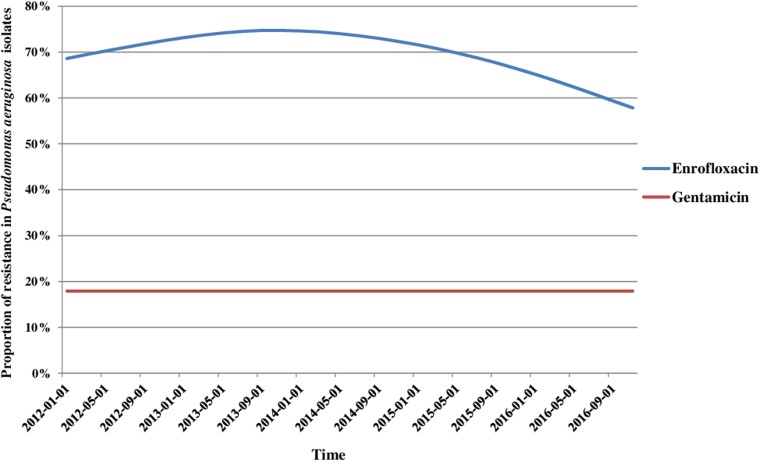
Trends for antimicrobial resistance in *Pseudomonas aeruginosa* isolates from dogs with otitis during the period 2012–2016, on a bimonthly time step.

We analysed the variations in resistance levels over the 2012–2016 period for all bacterial species presenting sufficient amounts of data, i.e. *S. pseudintermedius* (excluding cefovecin, fusidic acid and chloramphenicol) and *P. aeruginosa* isolates. Based on graphical analyses, no seasonal cycle was observed and the seasonal component in the model was never significant (data not shown). For *S. pseudintermedius*, resistance trends to erythromycin, gentamicin and trimethoprim-sulfamethoxazole were stationary from 2012 to 2016 (Fig. 1). The resistance trend to penicillin G was primarily stable, and then the resistance level increased from 62.5% (57.4–67.5) in March 2013 to 77.7% (70.4–85.0) in December 2016. By contrast, the resistance proportion to enrofloxacin increased at the beginning of the period, then decreased from 17.0% (13.5–20.4) in September 2013 to 7.8% (4.8–10.8) in December 2016. For *P. aeruginosa*, the resistance trend to gentamicin was stationary from 2012 to 2016 at 17.9% resistance (Fig. 2). The resistance trend to enrofloxacin varied: the resistance level first increased from 68.6% (55.3–82.0) in January 2012 to a maximum of 74.7% (68.2–81.2) in November 2013, and then decreased to 57.8% (51.1–64.5) in December 2016.

### Multidrug resistance

The MDR analyses were performed using five antibiotic classes for *P. mirabilis* and streptococci, and six classes for coagulase-positive staphylococci. Because only two antibiotics are commonly tested for *P. aeruginosa* by veterinary laboratories in France (enrofloxacin and gentamicin), the analysis for this bacterium was limited to these two antibiotics. The analysis of MDR included 4827 isolates for which complete records on resistance to the aforementioned antimicrobials classes were available (Table 2). Depending on the bacterial species studied, 20–30% of the isolates were susceptible to all antibiotic classes tested, except for *P. mirabilis* for which this proportion reached 63.8% (Table 4). The MDR proportions ranged between 11.8% for *P. mirabilis* and 20.7% for *S. pseudintermedius*. Only rare isolates were resistant to all antibiotic classes tested.

**Table 4.** Proportions of isolates (in % with 95% CI) that were pan-susceptible, resistant to one or two antibiotics from different classes, multidrug-resistant and resistant to all classes of antibiotics considered for analysis over the period 2012–2016

Bacterial species (total number of isolates)	Susceptibility patterns of isolates	Number of isolates	Proportion
*Staphylococcus pseudintermedius* (1066)	Pan-susceptible	248	23.3 (20.8–25.9)
	Resistant to one or two antibiotics classes	597	56.0 (53.0–59.0)
	Multidrug-resistant	221	20.7 (18.3–23.3)
	Resistant to the six classes of antibiotics considered	2	0.2 (0.0–0.7)
*Staphylococcus aureus* (212)	Pan-susceptible	52	24.5 (18.9–30.9)
	Resistant to one or two antibiotics classes	124	58.5 (51.5–65.2)
	Multidrug-resistant	36	17.0 (12.2–22.7)
	Resistant to the six classes of antibiotics considered	6	2.8 (1.0–6.1)
*Streptococcus* spp. (638)	Pan-susceptible	183	28.7 (25.2–32.4)
	Resistant to one or two antibiotics classes	373	58.4 (54.5–62.3)
	Multidrug-resistant	82	12.9 (10.4–15.7)
	Resistant to the five classes of antibiotics considered	4	0.6 (0.2–1.6)
*Pseudomonas aeruginosa* (1989)	Pan-susceptible	592	29.8 (27.8–31.8)
	Resistant to enrofloxacin and gentamicin	304	15.3 (13.7–16.9)
*Proteus mirabilis* (922)	Pan-susceptible	588	63.8 (60.6–66.9)
	Resistant to one or two antibiotics classes	225	24.4 (21.7–27.3)
	Multidrug-resistant	109	11.8 (9.8–14.1)
	Resistant to the five classes of antibiotics considered	6	0.7 (0.2–1.4)

## Discussion

### Aims and limits

The aim of our study was to determine the antimicrobial patterns of the most frequent bacterial causative agents of canine otitis isolated in France, and to describe the variations in resistance from 2012 to 2016. Using national data collected by the RESAPATH, we succeeded in assessing the levels of resistance for coagulase-positive staphylococci, *P. aeruginosa*, *P. mirabilis* and streptococci to antibiotics of relevance in veterinary and human medicine. From an epidemiological point of view, our findings address the knowledge gap regarding the resistance of *P. aeruginosa* and streptococci in animal health. To our best knowledge, this is the first study investigating resistance trends of the major causative agents of canine otitis in their full complexity, with methods ensuring non-linear trend detection. Using GAM models, we revealed the changes in AMR over the period 2012–2016.

With respect to the common genera and species of bacteria isolated from canine otitis, our findings are consistent with previous studies [5, 7, 11, 12, 17, 26, 27]: *S. pseudintermedius* is the most common ear pathogen isolated, followed by *P. aeruginosa*. However, the differences between sample selection and size, the method of strain collection, the bacterial population studied (commensal *vs.* pathogenic), the area of origin and the site of sampling (ears *vs.* dermatological sites including skin and mucosa) can explain variabilities between results and make data comparison between studies difficult. Only three studies had already explored the resistance of streptococci isolated from canine otitis or healthy ears [17, 27, 28]. Similarly to Bugden *et al*, we found high level of resistance to enrofloxacin (62.9%). However, contrary to their results and those of Hariharan *et al*, resistance to gentamicin was low in our study (3.3%) and consistent with results reported by Lyskova *et al*, whereas resistance to gentamicin in streptococci isolated in human medicine is almost non-existent [29]. In the present study, ceftiofur and gentamicin were the most effective antibiotics against *P. mirabilis*. To the best of our knowledge, the epidemiology of susceptibility to ceftiofur has not been investigated to date, even though one study in the literature reported *P. mirabilis* as a carrier of extended-spectrum *β*-lactamase genes and carbapenemases in France [30].

The major strength of our study was the availability of data regarding the susceptibility of canine otitis pathogens from an ongoing nationwide surveillance system for AMR in animal health: the RESAPATH network [19, 31]. Nevertheless, this study had several limitations due to potential selection bias, because laboratories join the RESAPATH on a voluntary basis and antibiograms rely on decisions taken by veterinarians during their veterinary practice. We could suppose that chronic otitis could prompt sample submission by veterinarians. The proportions of samples from previously untreated compared with treated dogs were also unknown and could potentially impact differently the results of resistance (i.e. through the selective antimicrobial pressure). The lack of information regarding antibiotic use in sampled dogs also limited us to confront directly in the models antibiotic use and resistance. In addition, it was not possible to differentiate first and subsequent sample submissions and we simply assumed that multiple sampling from the same dog did not occur frequently considering the cost of the analysis [32]. Although these biases can lead to a lack of representativeness and a misestimation of the levels of resistance, if biases do not vary over time, the observed resistance trends remain meaningful. Moreover, RESAPATH covers the full territory, thanks to its laboratory members which are implanted in all administrative regions in France. Not all French veterinary laboratories are members of RESAPATH (in 2015, 74 were members out of about 110 in the country), but RESAPATH receives the majority of the antibiograms performed in a clinical context in France [33]. Besides, an assessment of the network in 2015 concluded that the antibiograms from dogs collected by the RESAPATH were representative of the antibiograms from dogs performed in France [33]. Finally, epidemiological cut-off values were used in this study, but clinical breakpoints for antibiotics used in veterinary medicine would have been more reliable because they constitute more appropriate interpretative criteria for antibiogram results, but such criteria are not yet available in animal health in Europe [34].

### Public health interest

The high level of resistance of *S. pseudintermedius* isolates to penicillin G (68.5%) has been reported by other authors [12, 17, 27, 35] and mirror the situation in human clinical *S. aureus* isolates, where penicillin G resistance now exceeds 90%. In our study, despite the lack of further genetic investigations, methicillin resistance in *S. pseudintermedius* and *S. aureus* can be approximated by resistance to cefovecin (9.4%) and cefoxitin (10.6%), respectively. Here we clearly have an overestimation of the real levels of MRSP and MRSA, since these proportions aggregate resistant and intermediate isolates, while intermediate isolates should be confirmed as resistant by a molecular method. Nevertheless, our results indicated that the proportion of resistance to methicillin in staphylococci isolated from dogs with otitis in France is not negligible but remains below 10%. *S. pseudintermedius* is a much more prevalent pathogen in dogs (including for dogs presenting otitis infections) than *S. aureus*, suggesting that *S. pseudintermedius* is more adapted to colonise canine skin. In contrary, *S. pseudintermedius* remains a rare pathogen in humans and people in contact with dogs are more at risk to carry them [36, 37]. Guardabassi *et al*. highlighted that owners of dogs affected by chronic deep pyoderma often carry the same *S. pseudintermedius* strains as their dogs. Therefore, the zoonotic risk of transmission of MRSP between dogs with otitis and humans is considered limited for the general population, but might be higher for owners of dogs.

For *P. aeruginosa*, only two antibiotics (gentamicin and enrofloxacin) are routinely tested by French veterinary laboratories because only few antibiotics are authorised for veterinary use (aminoglycosides, fluoroquinolones and polymyxins). The high resistance of *P. aeruginosa* isolates from canine otitis to gentamicin (22.9%) found in this study is consistent with the findings of previous studies in Spain [38] and in Croatia [39]. Note that the resistance to gentamicin indicates resistance to all aminoglycosides used for the treatment of canine otitis in France (neomycin, framycetin, gentamicin). The high level of resistance of *P. aeruginosa* to enrofloxacin over the entire studied period (67.7%) is in accordance with results from many other studies [15, 16, 27, 29, 33–35] and is alarming, considering that fluoroquinolones are critically important antibiotics in human medicine. However, ciprofloxacin is considered to be a better marker of fluoroquinolones resistance for *P. aeruginosa* than enrofloxacin because resistance to ciprofloxacin indicates more precisely the resistance to fluoroquinolones [40]. But ciprofloxacin is not routinely tested by veterinary laboratories because it is restricted to human medicine. Therefore, the proportions of resistance to fluoroquinolones might have been overestimated in our study, but remain worrisome. In addition, we found that the proportion of *P. aeruginosa* isolates resistant to both gentamicin and enrofloxacin was 19.4% (17.7–21.2). This proportion is particularly worrying because *P. aeruginosa* is intrinsically resistant to many antibiotics including *β*-lactams, tetracycline, chloramphenicol, trimethoprim, kanamycin and quinolones [41, 42], and is also known for its ability to rapidly acquire additional resistances [38, 39]. The use of aminoglycosides and fluoroquinolones is also known to select *P. aeruginosa* isolates resistant to carbapenems, last resort antibiotics reserved for human medicine [43]. Thus, intrinsic and acquired resistance determinants potentially lead to therapeutic failures [43, 44].

### Parallel with antibiotic use and control measures

Our findings highlighted that resistance to fluoroquinolones has decreased since 2013 for both *S. pseudintermedius* and *P. aeruginosa*. Although the data regarding the use of antibiotics does not enable us to assess the current impact of use on AMR dynamics, one can assume that it contributed to the decrease in resistance observed in this study. In fact, between 2011 and 2016, the use of antibiotics in companion animals decreased overall and antibiotic exposure (Animal Level of Exposure to Antimicrobials indicators estimated by the French Agency for Veterinary Medicinal Products) dropped by 19.4% (all antibiotics considered) [45]. More specifically, fluoroquinolone exposure decreased by 56.6% [45]. However, the increase in penicillin resistance in *S. pseudintermedius* isolates does not coincide with the overall use of penicillins in pets, which dropped by 19.3% between 2011 and 2016. More accurate data on the specific use of antibiotics for canine otitis treatment are absolutely necessary to clearly document the relationship between use and resistance [46].

Besides, over the studied period, different measures related to AMR control might have also influenced resistance trends [19]: (i) the EcoAntibio plan, a National action plan to fight AMR in animal health, intended to promote the responsible use of antibiotics and effective from 2012 to 2016, (ii) the Law for the future of agriculture, food and forestry (October 2014) which sets a goal of reducing the consumption of CIAs by 25% by the end of 2016, and (iii) the Decree no. 2016–317 and Ministerial Order of 18 March 2016, which restrict the use of fluoroquinolones and last generations cephalosporins in animal health. Despite the uncertainty regarding the real period of effectiveness for these control measures, and a certain lag between implementations of measures and their potential effect on AMR, we observed that the drop in fluoroquinolone resistance began before the publication of law, ministerial orders and decrees that restricts the use of fluoroquinolones in veterinary medicine. We assume that the anticipation of new regulations and the EcoAntibio Plan contributed to more prudent use of antibiotics influencing the resistance trends to fluoroquinolones.

### Consequences for antimicrobial therapy

Our results underscore that dogs with otitis are a reservoir of resistant and multidrug-resistant bacteria. Knowledge of resistance levels and trends makes it possible to use antibiotics in animals in a careful and rational manner: our findings provide French veterinarians with comprehensive data to guide their initial empirical therapy of canine otitis. Guidelines are already available in France to assist veterinarians in their selection of the most appropriate antibiotics for initial therapy, but they do not provide specific recommendations according to the pathogen involved. Our results are essential to support and supplement them. The guidelines stress that knowledge of bacteria involved and antibiotic susceptibility patterns help in selecting an appropriate antibiotic for initial therapy of uncomplicated canine otitis. For otitis externa, French guidelines recommend to use topical treatment among the antibiotics available on the market (neomycin, framycetin, gentamicin, chloramphenicol, florfenicol, fusidic acid, trimethoprim-sulfamethoxazole or polymyxin B). Our findings characterised resistance to all of these antibiotics, except polymyxin B. We were not able to describe resistance to polymyxins because French veterinary laboratories use the disk diffusion method, which cannot clearly discriminate susceptible and resistant isolates due to poor diffusion of polypeptide antibiotics in agar [47]. Guidelines also stress the importance of carefully cleaning the ear canal [41] to limit inflammation. For otitis media, French guidelines point out the lack of data available on the susceptibility of bacteria involved. The first-line treatment consists in cleaning and flushing bulla [48]. Bacterial culture should be performed systematically even in the absence of visualisation of bacteria on cytology [41]. French guidelines also recommend the administration of systemic and topical antibiotics (among those available on the market). While awaiting the outcome of antibiograms, broad-spectrum antibiotics such as trimethoprim-sulfamethoxazole or gentamicin might be administered. Third-generation cephalosporins and fluoroquinolones remain second-line antibiotics in France: their use must be justified by isolation and antibiograms, except in emergency cases [49].

Our results support and clarify these recommendations. The present study documented staphylococcus (*S. pseudintermedius* and *S. aureus*), *P. aeruginosa*, streptococci and *P. mirabilis* as the most frequently isolated significant bacterial microorganisms from cases of otitis in dogs in France. Based on our results, if staphylococci are identified, gentamicin, fusidic acid or trimethoprim-sulfamethoxazole should be the appropriate antibiotic for therapy. Similarly, if streptococci are isolated, gentamicin and penicillin should be chosen. For *P. mirabilis*, gentamicin is appropriate as first-line treatment. Our findings also pointed out the difficulty of choosing appropriate therapy for *P. aeruginosa* without susceptibility testing. Resistance to gentamicin remains high and fluoroquinolones cannot be used as first-line antibiotics. Moreover, even though *P. aeruginosa* is uncommon as an initial pathogen, it frequently plays a major role in chronic otitis externa [38, 50], increasing therapeutic failure. To provide suitable treatment, veterinarians should also consider that the clinical presentation may influence the success of antibiotic therapy, as the presence of a purulent exudate will affect the efficacy of a particular antibiotic (gentamicin for instance [50]).

## Conclusion

This study provided an overall picture of resistance in staphylococci (*S. pseudintermedius* and *S. aureus*), *P. aeruginosa*, streptococci and *P. mirabilis* isolated from canine otitis. Our results are essential to support and supplement French guidelines. According to the level of resistance, while awaiting the outcome of antibiograms, broad-spectrum antibiotics such as gentamicin might be administered if staphylococci or streptococci are identified. Since 2013, resistance to fluoroquinolones has been on the decrease in both *P. aeruginosa* and *S. pseudintermedius* isolates; these decreases overlap with the implementation of the EcoAntibio plan, which promote the responsible use of antibiotics. For *P. aeruginosa*, 19.4% of isolates were resistant to both enrofloxacin and gentamicin, which is worrying because *P. aeruginosa* is intrinsically resistant to many antibiotics. As resistance to gentamicin remains high and fluoroquinolones cannot be used as first-line antibiotics, these findings point out the difficulty of choosing appropriate therapy for *P. aeruginosa* without susceptibility testing.

## Data Availability

The data used for this study were obtained from the RESAPATH network. Conditions of approval (respecting the anonymity of farms and laboratories) do not allow us to distribute or make data available directly to other parties.
